# The direct cost of dialysis supported by families for patients with chronic renal failure in Ouagadougou (Burkina Faso)

**DOI:** 10.1186/s12882-022-02847-y

**Published:** 2022-06-23

**Authors:** Amadou Oury Toure, Mamadou Dioulde Balde, Aissatou Diallo, Sadan Camara, Anne Marie Soumah, Alpha Oumar Sall, Karifa Kourouma, Bienvenu Salim Camara, Fadima Yaya Bocoum, Seni Kouanda

**Affiliations:** 1 Center for Research in Reproductive Health/Cellule de recherche en santé de la reproduction en Guinée (CERREGUI), Conakry, Guinea; 2 National Center for Training and Research in Rural Health in Maferinyah Guinea /Centre national de formation et de recherche en santé rurale à Maferinyah Guinée, Maferinyah, Guinea; 3 Institut de Recherche en Sciences de la Santé (IRSS) Burkina Faso, Ouagadougou, Burkina Faso; 4 African Institute of Public Health (IASP) Burkina Faso/Institut Africaine de Santé Publique Burkina Faso, Ouagadougou, Burkina Faso

**Keywords:** Direct cost, Dialysis, Management, Chronic renal failure, Burkina Faso

## Abstract

**Background:**

Chronic renal failure can lead to dialysis and/or a kidney transplant in the final stage*.* The number of patients under dialysis has increased considerably in the world and particularly in sub-Saharan Africa. Dialysis is a very expensive care. This is the reason why this study on the costs of dialysis management was initiated in Burkina Faso. The objective of the study is to determine the direct medical and non-medical costs of managing chronic renal failure among dialysis patients in Ouagadougou in 2020.

**Methods:**

An analytical cross-sectional study was conducted. Data were collected in the hemodialysis department of three public university hospitals in Ouagadougou, Burkina Faso. All dialysis patients with chronic renal failure were included in the study. Linear regression was used to investigate the determinants of the direct medical and non-medical cost of hemodialysis.

**Results:**

A total of 290 patients participated in this study, including children, adults, and the elderly with extremes of 12 and 82 years. Almost half of the patients (47.5%) had no income. The average monthly total direct cost across all patients was 75842 CFA or US$134.41.The average direct medical cost was 51315 CFA or US$90.94 and the average direct non-medical cost was 24 527 CFA or US$43.47. Most of the patients (45.2%) funded their hemodialysis by their own source.

The multivariate analysis showed that the presence of an accompanying person during treatment, residing in a rural area, ambulatory care, use of personal cars, and treatment at the dialysis center of Yalgado Teaching Hospital were associated with higher direct costs.

**Conclusion:**

The average cost of dialysis services borne by the patient and his family is very high in Burkina Faso, since it is 2.1 times higher than the country's minimum interprofessional wage (34664 CFA or US$61.4). It appears that the precariousness of the means of subsistence increases strongly with the onset of chronic renal failure requiring dialysis. Thus, to alleviate the expenses borne by dialysis patients, it would be important to extend the government subsidy scheme to the cost of drugs and to promote health insurance to ensure equitable care for these patients.

## Background

Chronic kidney disease is a serious, disabling, and fatal disease. It is nowadays a real public health problem in both developed and emerging countries [1]. Its prevalence and incidence are constantly increasing, mainly due to the aging of the population and the increase of metabolic pathologies that damage the kidneys, including diabetes and hypertension [2]. The number of patients under dialysis has increased dramatically worldwide, including sub Saharan Africa [3].

In developed countries, the costs of chronic kidney disease management are generally supported through health insurance mechanisms; these costs vary according to the treatment method [4–6]. In France, the overall cost supported by the health insurance system amounted to 2.1 billion euros in 2005 [7]. While in Switzerland the treatment of chronic kidney disease by hemodialysis costs approximately €80000 per patient per year [8]. In the Netherlands the annual non-healthcare cost has (costs related to lost productivity) been estimated at €8284 (standard deviation (SD): €14266) for transplant patients and €23,488 (SD: €39434) for dialysis patients [4]. In South Korea, the average annual cost of hemodialysis (Cost of care delivery, cost of drugs and cost of patient transportation) was €34554 per patient [9]. In the United States of America, this cost represents nearly 7.1% of the total Medicare costs [10]

A comparative study in Algeria between the private and public sectors on the evaluation of hospital costs for the management of chronic end-stage renal disease reported that the average cost of care for chronic renal failure is significantly higher in private than in public facilities, with 1313.4 and 2800.11 US dollars respectively [11]. Direct payment (especially fee-for-service) is the most common method of payment in Africa and is a real barrier to access to care because it reduces utilization of services [3].

Based on previous studies on the costing of chronic kidney disease management, evidence has been generated. This evidence highlighted the different costs borne by health insurance, patients, and public and private facilities in Africa [2, 4, 7, 12]. However, due to the fact that there are few studies on this topic in Africa, particularly in the case of Burkina Faso, where renal dialysis is subsidized by the government, no data have been reported in the literature on the direct medical and non-medical costs borne by households. The lack of knowledge of these costs can lead to inequality and inaccessibility of care for patients.

The objective of this study was to determine the direct cost of managing chronic renal failure among dialysis patients, from the perspective of patients and families in Ouagadougou in 2020. It is related to direct medical and non-medical costs by focusing on the estimation of different costs, patient revenue sources and factors affecting the cost of management.

### Study setting

Burkina Faso is located in the Sahelian region of West Africa. It has an area of 274,200 km2. The country is administratively divided into 13 regions, 45 provinces, 351 communes, and 8000 villages. It has an agro-pastoral vocation. Agriculture and livestock employ 86% of the active population and provide 30% of Gross Domestic Product (GDP) and 80% of export earnings. The population growth rate is 3.1% [13, 14]. According to the Human Poverty Index in 2009, Burkina Faso was ranked 131st out of 135 countries with an estimated GDP per capita of US$1124. The majority of its population, 81.2%, lives below the poverty line (US$2 per day) and 46% below the national poverty line [15].

The Burkinabe population is predominantly young, with 59% under the age of 20 [14].

The health system in Burkina Faso has two organizations (administrative and operational). The administrative organization includes the central, intermediate (13 Regional Health Directorates), and peripheral (63 Health Districts) levels; the operational organization includes the first level, which is composed of the Social Promotion Health Centers and the Medical Centers, the second level which includes the District hospital, the Regional Hospital Centers and the third level which is the University Hospital Center [16].

## Methods

### Study design and period

The present study was cross-sectional and analytical, based on individual interviews with patients in the health facilities. Data collection was carried out from July to August 2020.

### Study sites

This study took place in the three public hemodialysis departments of Ouagadougou, namely those of Yalgado Ouedraogo, Tingandogo, and Bogodogo Teaching Hospitals. These departments were chosen because they are the main reference management centers for renal pathologies in the whole country.

### Study population

Our study included all patients with chronic end-stage renal disease who had completed at least one month of dialysis, whether or not they were hospitalized in one of the three university hospitals (Yalgado Ouedraogo, Tingandogo and Bogodogo).

The criteria for non-inclusion in this study were:

- Not giving consent to participate in the study,
- Being a patient under dialysis but has lost his/her invoice for medication, examinations, hospitalization, or consultation,
- Being a patient under dialysis with a deteriorated general condition, and with no present accompanying person,
- Being a patient with unusable data (medical records, health booklet)
- Being patient with a mental disorder.

### Study variables

These include a dependent variable and independent variables.

The average monthly direct cost of dialysis is the dependent variable. It is a quantitative variable, expressed in CFA francs. It is the sum of direct medical and non-medical cost. The direct medical cost is the sum of consultation cost, drug costs, laboratory test cost, imaging cost, and hospitalization cost. The direct non-medical cost includes the cost of transportation, food, water, beverages, telephone credits, and fuel. The 17 independent variables included age, gender, education level, marital status, occupation, origin, source of care funding, patient income level, comorbidity, duration of treatment, number of hemodialysis sessions per week, home-hospital distance, means of travel, household size, name of the university hospital, patient accompaniment, residence, and hospitalization.

### Data Collection

Data were collected using a structured questionnaire. The data collection team comprised the principal investigator three medical students. They were trained for two days on: ethical aspects, the definition of direct medical and non-medical cost, filling in the expenditure tables, and post-dialysis complications. The questionnaire was then pre-tested for validation. Cost collection was based on evidence of receipts and/or invoices. The interviews were conducted in French and in three local languages of Burkina Faso (More, Foulfoulde, Dioula).

### Data analysis

Data were entered using Epidata software, then exported to Stata 15 software for analysis. Descriptive statistics were performed for socio-demographic characteristics. Pearson chi-square and Fisher's exact test were used to compare categorical variables. The Kruskal-Wallis test was used for continuous quantitative variables with skewed distributions to compare group means and categories. We used linear regression to search for determinants of direct medical and non-medical cost of dialysis has a significance level set at p<0.05. Coefficients were reported with p-values and 95% confidence intervals. The modeling was done in several steps. First, we performed a Mackinon's Fe test to check the linear versus a logarithmic form of the variables. This test oriented us towards the log. Thus, we logged the dependent variable (direct medical and non-medical cost) and all quantitative variables that did not have a normal distribution. A univariate top-down regression allowed us to select the variables likely to be included in the model. This allowed us to exclude some variables.

The multi-collinearity test was performed between the independent variables that were significantly associated with the dependent variable in the univariate regression. All the multicollinearity tests were less than 10, suggesting the absence of collinearity between the variables. Subsequently, we performed a multivariate analysis by progressively introducing the independent variables that had been significantly associated with the dependent variable in the univariate analysis and selected after the multicollinearity test. In the same framework of analysis, the multivariate regression model was estimated using the robust Ordinary Least Squares estimator to correct potential heteroscedasticity biases that could affect the normal distribution of residuals and create statistical biases at the same time. Each time we introduced a new variable, we checked the number of observations, the p-value, and the R-squared of the model. The Ramsey reset test was performed to check the specification of the model. Finally, we proceeded to the recovery of the residuals, then the verification of their normalities by the non-parametric test of Kolmogorov Smirnov, then the test of Anderson-Darling, and a graphic test for an illustration.

## Results

Table 1 shows that 290 patients participated in the study including children, adolescents, adults, and the elderly. The mean age of the study population was 44.3 ±14 years with extremes of 12 and 82. The age range of 34 to 44 years was the most frequent. Females represented 40.3%. The most represented socio-professional category was the unemployed (26.2%), followed by housewives (18.3%). Those with no income represented 47.5%. Patients with secondary education represented 42.0%.

**Table 1:** Socio-demographic and socioeconomic characteristics

	Number	%
**Age (Years)**		
12 - 22	13	4,5
23 - 33	60	20,7
34 -44	77	26,6
45 -55	73	25,2
56 - 66	49	17
67 and over	18	6,2
**Sex**		
Male	173	59,7
Female	117	40
**Occupation**		
Public sector	50	17,2
Private sector	35	12,1
Self employement	37	12,8
Students/pupils	18	6,2
Housewives	53	18,3
Retired	21	7,2
Unemployed	76	26,2
**Marital status**		
Singles	6	2,1
Married	186	64,1
Divorced / Widowed	61	21,0
Concubinage	37	12,8
**Education level**		
Not schooling	50	17,2
Primary	46	15,9
Secondary	122	42,1
University	52	18
Schooling in arabic language	20	7
**Income periodicity**		
No income	139	47,6
Daily	33	11,4
Monthly	102	35,2
Trimestrial	12	4,1
Annual	5	1,7
**Level of income**		
No income	139	47,9
Lower than the minimum salary (34 664 Fcfa)	32	11,0
34 664 to 100 000 Fcfa	31	10,7
100 000 to 300 000 Fcfa	66	22,8
300 000 to 500 000 Fcfa	15	5,2
500 000 Fcfa and More	7	2,4

The mean age of the study population was 44.3 ±14 years with extremes ranging from 12 to 82

### Frequency of dialysis per week, monthly direct cost and source of funding

More than half of the patients (55.86%) had an average of 2 dialysis sessions per week [95% CI= 1.628126; 1.764978], contrary to the usual recommendations of 3 sessions per week in end-stage renal disease.

Overall, the monthly average of direct cost was 75842 CFA (US$ 134.41) ranging from 2800 CFA (US$ 5.00) to 1117200 CFA (US$ 180.06). In the analysis of direct medical cost borne by patients (including consultation, para-clinical examinations, hospitalization, medication, and consumables), the average monthly cost was 51315 CFA (US$ 90.94) (p-values= 0.000), with a variation depending on the number of sessions performed per week Table 2. As for the average direct non-medical cost (transportation, food, fuel, and miscellaneous), it amounted to 24527CFA (US$ 43.47) with significant variations from a hospital to another (p-values= 0.000). The direct non-medical cost represented half of the total direct cost.

**Table 2:** Frequency of dialysis per week, monthly direct cost and source of funding

**Dialysis sessions per week**			
Number of sessions	%	Standard-Error	IC 95%
1	37,24	2,8	[31,83 ; 42,98]
2	55,86	2,9	[50,06 ; 61,50]
3	6,90	1,4	[4,48 ; 10,47]
**Monthly direct costs of dialysis by number of sessions**			
Average cost of dialysis by number of sessions per week	Average cost in FCFA	Standard-Error	IC 95%
1	59507.37	3462.735	[52691.99 ; 66322.75]
2	77 829.265	4821.571	[68339.42 ; 87319.11]
2	147 953.9	52779.27	[44073.41 ; 251834.4]
**Total**	75842.121		[66380 ; 85304]
Non parametric test of Kruskall – Wallis	**Chi 2**	9.995	
	***p*-value**	0.0068	
**Sources of funding**			
		Number	%
Himself		131	45,2
Relatives		95	32,8
Childrens		42	14,5
Friends		14	4,8
Others		7	2,4
Insurance		4	1,4
Social assistance		4	1,4
Employer and collaborators		1	0,3

As Table 2 shows, 45.2% of patients paid for their dialysis sessions from their own funds. Financial support from parents represented 32.8% and from children 14.5%. Other important funding sources were friends, colleagues, and associations. Private insurance coverage was only 1.4%.

The costs of care disaggregated by patient socio-demographic characteristics are presented in Table 3.

**Table 3:** Cost breakdown by patient socio-demographic and socio-economic characteristics

Socio-demographic and socio-economic characteristics	Average cost in FCFA	Standard-Error	IC 95%
**Age (Years)**			
12 - 22	61 528.769	12782.49	[36370.19 ; 86687.35]
23 - 33	57 686.8	4196.753	[49426.72 ; 65946.88]
34 - 44	80 013.273	14304.68	[51858.71 ; 108167.8]
45 - 55	75 418.685	8109.292	[59457.93 ; 91379.44]
56 - 66	90 155.245	9512.978	[71431.74 ; 108878.8]
67 and over	91 607.778	13385.04	[65263.26 ; 117952.3]
Non parametric test of Kruskall – Wallis	**Chi 2**	12.272	
	***p*-value**	0.0312	
**Sex**			
Female	75170.53	5443.334	[64456.93 ; 85884.13]
Male	76296.318	7181.621	[62161.4 ; 90431.23]
Non parametric test of Kruskall – Wallis	**Chi 2**	0.501	
	***p*-value**	0.4790	
**Education level**			
Not schooling	53 777.54	3875.048	[46150.64 ; 61404.44]
Primary	66 178.87	10273.65	[45958.21 ; 86399.53]
Secondary	76 524.492	4752.949	[67169.71 ; 85879.28]
University	108 125.46	21506.2	[65796.82 ; 150454.1]
Schooling in arabic language	65 129.9	9784.239	[45872.5 ; 84387.3]
Non parametric test of Kruskall – Wallis	**Chi 2**	14.940	
	***p*-value**	0.0048	
**Occupation**			
Public sector	86 548.92	6925.858	[72917.4 ; 100180.4]
Private sector	113 863.63	31054.88	[52741.21 ; 174986]
Self employement	59 898.73	11736.62	[36798.65 ; 82998.81]
Students/pupils	64 805.056	11588.57	[41996.36 ; 87613.75]
Housewives	66 236.094	4608.069	[57166.46 ; 75305.72]
Unemployed	53 692.742	4380.696	[45070.63 ; 62314.86]
Invalid	82 206.857	22748.01	[37434.07 ; 126979.6]
Retired	120 059.4	18388.27	[83867.46 ; 156251.3]
Other	51 805.714	9391.692	[33320.92 ; 70290.5]
Non parametric test of Kruskall – Wallis	**Chi 2**	36.720	
	***p*-value**	0.0001	
**Place of residence**			
Urban	70703.13	3597.831	[63621.85 ; 77784.4]
Rural	101117.6	22117.3	[57586.16 ; 144649]
Non parametric test of Kruskall – Wallis	**Chi 2**	5.829	
	***p*-value**	0.0158	
**Income periodicity**			
No income	61372.96	3246.261	[54983.64 ; 67762.27]
Daily	49349.09	4907.64	[39689.84 ; 59008.34]
Monthly	102541.2	12177.23	[78573.94 ; 126508.5]
Trimestrial	94775.83	20038.14	[55336.64 ; 134215]
Annual	59942.2	8003.258	[44190.14 ; 75694.26]
Non parametric test of Kruskall – Wallis	**Chi 2**	23.672	
	***p*-value**	0.0001	
**Level of income**			
Lower than the minimum salary (34664 Fcfa)	59 457.784	2 811.552	[53924.07 ; 64991.5]
34664 to 99000 Fcfa	92 143.323	15 710.14	[61222.52 ; 123064.1]
100000 to 299 000 Fcfa	101 838.86	17 267.83	[67852.21 ; 135825.5]
300000 to 499 000 Fcfa	90 221.2	16 652.64	[57445.37 ; 122997]
500 000 Fcfa and More	127 972.57	21 434.27	[85785.51 ; 170159.6]
Non parametric test of Kruskall – Wallis	**Chi 2**	23.265	
	***p*-value**	0.0001	

Breakdown of costs according to patients' socio-demographic and socio-economic characteristics

The breakdown of costs according to the socio-demographic and economic characteristics of the patients (age, sex, occupation, education, place of residence) showed that the difference was statistically significant (P<0.05) in the components: age, occupation, education and place of residence.

The financial burden is higher for people aged 67 and over. Indeed, the elderly spend 1.5 times more than young people aged 12 to 22 years (respectively 91,607,778 for people aged 67 years and over and 61,528,769 for children and young people aged 12 to 22 years). This difference was statistically significant with P = 0.03.

In the occupational component, retirees and private employees had a higher average expenditure followed by the disabled. This result was statistically significant at P= 0.00.

Patients with a higher level of education spend twice as much for their dialysis as those with no education (108,125.46 FCFA and 53,777.54 FCFA respectively) and this result was statistically significant P= 0.00.

However, the variation by gender does not show a statistically significant difference between men and women.

### Factors associated with the direct cost of managing dialysis patients

Table 4 on univariate linear regression shows that occupation, income, companionship for care, distance, and patient age are statistically associated with dialysis management direct cost. However, after the multivariate analysis, companionship, residence, mode of care, means of travel, and place of care were independently associated with the dialysis management direct cost. Indeed, patients living in rural areas spent 33% more money on hemodialysis than those living in urban areas (coefficient: 0.338; 95% CI [0.108; 0.567]. As for the mode of medical follow-up, patients who were not hospitalized spent 50% less money on their hemodialysis than hospitalized patients (coefficient: -0.513; IC 95% [-0.740; -0.286]. Patients using motorcycles to get to the place of care spent 48% less money than patients who traveled by private cars. Those who used means other than taxi and motorcycle spent more than one time less than those who traveled by personal car. Patients who received care at the Bogodogo Teaching Hospital spent 49% less than those who received care at the Yalgado Ouedraogo Teaching Hospital.

**Table 4:** Factors associated with the monthly direct cost of dialysis

Log of direct costs	Coefficients	Standard-Error	***p***-value	IC 95%
**Income (CFA)**				
Under Guaranteed minimum wage 34,664(ref)	0,000	.	.	.
34,664 - 99,000	0,148	0,164	0,366	[-0,174 ; 0,471]
100,000 - 299,000	0,109	0,157	0,488	[-0,200 ; 0,418]
300,000 - 499,000	0,195	0,231	0,401	[-0,261 ; 0,651]
500,000 and more	0,218	0,299	0,466	[-0,370 ; 0,806]
**Distance (**km)				
Under 5 (ref)	0,000	.	.	.
5 - 9	0,197	0,138	0,155	[-0,075 ; 0,468]
10 and more	0,016	0,131	0,906	[-0,243 ; 0,274]
**Accompanying the patient**				
With companion (ref)	0.000	.	.	.
Without companion	-0,171	0,087	0,051	[-0,342 ; 0,001]
**Level of education**				
Not schooling (ref)	0,000	.	.	.
Elementary	0,088	0,125	0,483	[-0,158 ; 0,334]
Secondary	0,183	0,123	0,141	[-0,061 ; 0,426]
High education/ university	0,143	0,161	0,374	[-0,174 ; 0,460]
Arabic schooling	0,112	0,156	0,474	[-0,195 ; 0,418]
**Occupation**				
Public sector (ref)	0,000	.	.	.
Private sector	0,212	0,144	0,144	[-0,073 ; 0,497]
Self employement	-0,251	0,177	0,157	[-0,600 ; 0,097]
Student/pupil	0,007	0,238	0,976	[-0,462 ; 0,477]
Housewife	-0,016	0,198	0,937	[-0,405 ; 0,374]
Unemployed	-0,131	0,181	0,471	[-0,488 ; 0,227]
Invalid	0,083	0,293	0,778	[-0,494 ; 0,660]
Retired	-0,032	0,194	0,870	[-0,414 ; 0,350]
Other	-0,275	0,268	0,305	[-0,803 ; 0,252]
**Location**				
Urban (ref)	0,000	.	.	.
Rural	0,338	0,116	0,004	[0,108 ; 0,567]
**Marital status**				
Married polygamist (ref)	0,000	.	.	.
Married monogamous	-0,115	0,123	0,350	[-0,358 ; 0,127]
Divorced	-0,205	0,164	0,212	[-0,528 ; 0,117]
Single	-0,295	0,159	0,064	[-0,607 ; 0,018]
Concubinage	-0,083	0,277	0,766	[-0,628 ; 0,463]
**Hospitalization**				
Hospitalized (ref)	0.000	.	.	.
Non-hospitalized	-0,513	0,115	0,000	[-0,740 ; -0,286]
**Number of dialysis sessions**				
1 (ref)	0,000	.	.	.
2	-0,081	0,160	0,612	[-0,395 ; 0,233]
3	0,097	0,230	0,672	[-0,355 ; 0,550]
**High blood pressure**	0,152	0,247	0,538	[-0,334 ; 0,638]
**Diabetes**	-0,167	0,132	0,207	[-0,426 ; 0,093]
**Transports**				
Private car (ref)	0,000	.	.	.
Taxi	-0,206	0,150	0,171	[-0,503 ; 0,090]
Motorcycle	-0,482	0,102	0,000	[-0,684 ; -0,280]
Others	-1,182	0,274	0,000	[-1,722 ; -0,641]
**Teaching Hospital**				
Yalgado Ouédraogo (ref)	0,000	.	.	.
Bogodogo	-0,492	0,176	0,006	[-0,838 ; -0,146]
Tingandogo	-0,217	0,115	0,061	[-0,444 ; 0,010]
**Lenght of care**	0,008	0,044	0,851	[-0,078 ; 0,094]
**Complications of dialysis**	0,074	0,278	0,789	[-0,473 ; 0,622]
**Hyperuricemia disease**	-0,015	0,174	0,931	[-0,358 ; 0,327]
**Sickle cell disease**	0,253	0,351	0,471	[-0,438 ; 0,945]
**Household size**	0,009	0,011	0,390	[-0,012 ; 0,030]
**Sex**				
Female (ref)	0,000	.	.	.
Male	-0,050	0,099	0,613	[-0,246 ; 0,146]
**Age** (years)				
12 - 22 (ref)	0,000	.	.	.
23 - 33	0,181	0,217	0,404	[-0,246 ; 0,608]
34 - 44	0,248	0,242	0,307	[-0,229 ; 0,724]
45 - 55	0,033	0,244	0,894	[-0,448 ; 0,514]
56 - 66	0,127	0,255	0,619	[-0,376 ; 0,631]
67 and more	-0,095	0,282	0,737	[-0,651 ; 0,461]
**Constant**	6,248	2,434	0,011	[1,453 ; 11,043]

## Discussion

Our study on the evaluation of the dialysis management direct cost reveals that the financial burden on patients under dialysis remains high despite the subsidy from the Burkinabe government.

### Direct costs

The monthly average direct cost borne by patients is 75842 CFA (134.41 US$); this equal to a yearly average cost of 910104 CFA (1613 US$). Compared to the guaranteed minimum wage 34664 CFA, this average cost remains very high in the Burkinabe context. Indeed, this direct cost has a non-medical component outside the health facility (transportation, catering, communication costs, etc.) which constitutes half of the cost paid by the patient. In addition, direct medical costs such as examinations, specialist drugs (diuretics) are very high, especially in private drug stores. This cost, therefore, constitutes a financial barrier to access patients’ access to care. A study carried out in the city of Kinshasa (Democratic Republic of Congo) in a non-subsidized context reported an annual average cost of hemodialysis of US$28,280, i.e. 22 times higher than the direct cost in Burkina Faso [17]. This low direct cost of hemodialysis found in our context compared to that of the Democratic Republic of Congo could be explained by several factors such as the existence of a subsidy for dialysis in Burkina Faso, the fact that patients do not perform all check-ups and also-they select the drugs to be purchased because of their limited resources.

Other studies have estimated both direct and indirect cost of dialysis. For example, in Iran the estimated total cost of each hemodialysis session was about 74 US$ and the annual cost per patient was 11549 US$ [12]. A study reported that in Brazil the average total cost per patient-year is US$ 28570 for hemodialysis [5]. In Saudi Arabia, the average total cost per hemodialysis session was calculated to be 297 US$ [1,114 Saudi riyals (SR)], and the average total cost of dialysis per patient per year was 46 332 US$ (173784 SR) [18].

### Source of funding

Our study reports that self-funding (45.5%), parental financing, and financial support from children remain the main sources of funding for patients. The low socio-economic level, patients’ disability, the lack of payment mechanisms through health insurance or social action could explain this result. In fact, few patients in Burkina Faso have health insurance or social action mechanisms. This could be explained by patients' lack of information about health insurance and the rarity of these mechanisms in our context. The absence of these indirect payment mechanisms can lead to inequality of access to care.

The breakdown of costs according to the socio-demographic and socio-economic characteristics of the patients showed that patients living in rural areas spent more than those in urban areas. This could be explained by the cost of transportation related to the distance travelled by these patients.

Retirees and private sector employees had a higher average expenditure than other occupations, respectively 120,059.4 and 113,863.63 CFA francs. This can be explained on the one hand by the age of retired people which leads to a fragility of the organism and which also exposes them to chronic pathologies. On the other hand, private sector employees benefit more from health insurance than others and probably have a higher salary.

### Main associated factors

The place of residence (urban or rural), the type of follow-up, the means of travel, and the place of care (university hospital) were the factors associated with the direct cost of dialysis. Indeed, the presence of a companion during care, residence in a rural area, ambulatory follow-up, use of personal cars, and care in the dialysis center of the Yalgado Teaching Hospital had a significant association with higher direct cost. Other studies in the Democratic Republic of Congo and China found comorbidity and residence environment as factors significantly associated with the cost of dialysis [6, 17].

### Strengths and limitations of the study

This is the first study in Burkina Faso to examine the direct cost of dialysis management by patients. It is also one of the few studies that have analyzed the sources of funding for the direct cost of dialysis by patients. As limitations, this study was circumscribed to Ouagadougou public hemodialysis department. Thus, private and the countryside hemodialysis centers were not covered by the research.

### Implications for research and practice

The new knowledge gained from this study contributes to a better understanding of the cost of chronic kidney disease management in Ouagadougou. The results of this study will help to guide advocacy and actions for improving access to chronic kidney disease services in Burkina Faso. A larger study including private and countryside hemodialysis centers will provide more important data for a holistic understanding of the cost for managing chronic kidney disease in Burkina Faso. Nonetheless, establishing a functional health insurance system is a priority to alleviate health expenses for patients under dialysis in this country.

## Conclusion

The average cost of dialysis services borne by patients and their families is very high in Burkina Faso (34664 CFA or 61.4 US$), representing 2.1 times the guaranteed minimum interprofessional wage. This study revealed that a chronic renal failure requiring dialysis greatly increases the precariousness of patients' livelihoods. Therefore, to alleviate these patients’ expenses, it is paramount to extend the state subsidy package to the cost of drugs and promote health insurance for equitable cares.

## Data Availability

The data supporting the results of this study are available from [Amadou Oury Toure] but restrictions apply to the availability of these data and are therefore not publicly available, as our research group is working on further analyses using the same data which will then be submitted for publication. However, these data are available upon reasonable request to the corresponding author [Amadou Oury Toure].
